# Supplementary material for: Causal Relationship between Aging and Anorexia Nervosa: A White-Matter-Microstructure-Mediated Mendelian Randomization Analysis
Source: Biomedicines. 2024 Aug 16;12(8):1874. doi: 10.3390/biomedicines12081874 (PMC11351342; doi:10.3390/biomedicines12081874)
Supplement: Supplementary file 1 [file biomedicines-12-01874-s001.zip › Supplementary Figure/Figure S8/Leaveoneout plot of MR analysis for TL to ubm-b-1747.pdf]

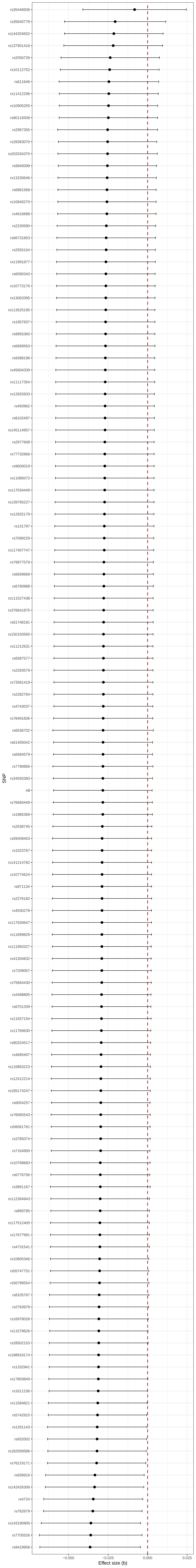
rs35446936
rs35640778
rs144204502
rs137901416
rs2056726
rs10112752
rs611646
rs11412296
rs10905255
rs80116508
rs2967355
rs28363070
rs202034370
rs9940099
rs13230646
rs6881568
rs10840270
rs4616688
rs2230590
rs66731853
rs2555104
rs11991877
rs6590343
rs10773176
rs13062095
rs113525195
rs1957937
rs9955360
rs6669563
rs9398196
rs45604339
rs11117354
rs12925933
rs450962
rs8102497
rs145114957
rs2977608
rs77732866
rs9600019
rs11085072
rs117034449
rs139795227
rs12932179
rs131797
rs7099229
rs117407747
rs79977579
rs6659669
rs6790988
rs111527438
rs376641875
rs61748181
rs150150565
rs11212631
rs6587577
rs2293579
rs73581419
rs2282764
rs4743037
rs78491606
rs6536702
rs61405042
rs6584579
rs7790856
rs34550383
All
rs76666449
rs1985369
rs2538745
rs59409453
rs1023767
rs141214782
rs10774624
rs871134
rs2276182
rs4530278
rs117630647
rs11699829
rs111950327
rs41304832
rs7209057
rs75664430
rs4498805
rs6751209
rs11557154
rs11769630
rs80324517
rs4695407
rs116863223
rs12412214
rs185174247
rs6054257
rs76065543
rs56061761
rs3785074
rs7164950
rs10768683
rs6776756
rs3891167
rs112394943
rs869785
rs117512405
rs17677991
rs4731541
rs10805346
rs55747751
rs56799554
rs8105767
rs2763979
rs16978028
rs11579626
rs28502153
rs188918174
rs1332941
rs17803849
rs1611236
rs11584821
rs5742915
rs1291143
rs932002
rs182059586
rs76219171
rs939916
rs142426306
rs4724
rs762679
rs143190905
rs7705526
rs9419958
SNP
-0.050
-0.025
0.000
0.025
Effect size (b)
